# Renal Function, Atrial Cardiopathy, and Their Joint Association with Mortality in the General Population

**DOI:** 10.3390/jcm15010122

**Published:** 2025-12-24

**Authors:** Tarek Zaho, Mai Z. Soliman, Mohamed A. Mostafa, Ahmed E. Shatta, Mohamed A. Attia, Menna S. Elbadawy, Richard Kazibwe, Elsayed Z. Soliman

**Affiliations:** 1 Epidemiological Cardiology Research Center (EPICARE), Department of Cardiovascular Medicine, Wake Forest School of Medicine, Winston Salem, NC 27157, USA; tarek.zaho@advocatehealth.org (T.Z.); msoliman@wakehealth.edu (M.Z.S.); mmostafa@wakehealth.edu (M.A.M.); ahmed.shata@live.com (A.E.S.); mohamedashraf511@gmail.com (M.A.A.); menna.elbadawy@yahoo.com (M.S.E.); 2 Section on Hospital Medicine, Department of Medicine, Wake Forest School of Medicine, Winston Salem, NC 27157, USA; richard.kazibwe@advocatehealth.org

**Keywords:** atrial cardiopathy, renal impairment, P-wave axis, deep terminal negativity in V1, prolonged P-wave duration, NAHNES-III

## Abstract

**Background**: Both atrial cardiopathy and impaired renal function are independently associated with increased mortality, but their interrelationship and combined impact remain uncertain. **Methods**: We analyzed 6573 participants from NHANES-III (mean age 57 years; 50.5% women; 74.6% White) with available electrocardiograms (ECGs). Estimated glomerular filtration rate (eGFR) was calculated using the CKD-EPI equation. Atrial cardiopathy was defined by any of the following ECG markers: abnormal P-wave axis (<0° or >75°), deep terminal negativity in lead V1 (>100 µV), or prolonged P-wave duration in lead II (>120 ms). Participants with eGFR <15 mL/min/1.73 m^2^ or major ECG abnormalities were excluded. Logistic regression assessed the association between impaired renal function (eGFR < 45 vs. ≥45 mL/min/1.73 m^2^) and atrial cardiopathy. Cox models evaluated independent and joint associations of impaired renal function and atrial cardiopathy with all-cause mortality. **Results**: About 47.9% (*n* = 3151) had atrial cardiopathy at baseline, of whom 161 (4.7%) had impaired renal function. Impaired renal function was associated with higher odds of atrial cardiopathy (OR 1.44; 95% CI 1.16–1.78). Over a median follow-up of 18.1 years, 3076 deaths occurred. Compared with participants without either condition, those with both had the highest mortality risk (HR 1.68; 95% CI 1.46–1.94), exceeding risks from atrial cardiopathy alone (HR 1.10; 95% CI 1.02–1.18) or impaired renal function alone (HR 1.42; 95% CI 1.18–1.70; *p* = 0.011 for interaction). **Conclusions**: Impaired renal function is associated with a greater prevalence of atrial cardiopathy. Their coexistence exerts a synergistic effect, substantially amplifying mortality risk beyond either condition alone.

## 1. Introduction

Atrial cardiopathy is defined as a spectrum of structural, architectural, contractile, or electrophysiological changes affecting the atria, which have the potential to produce clinically relevant manifestations [1,2]. Observational studies have shown that electrocardiographic and imaging markers of atrial dysfunction are associated with, for example, stroke, heart failure, and mortality, even in the absence of clinical arrythmia such as atrial fibrillation (AF), suggesting that atrial cardiopathy has a prothrombotic and hemodynamically adverse atrial foundation that precedes clinically recognized arrhythmia [3,4]. With the absence of therapeutic modalities directed towards atrial cardiopathy, identifying conditions that contribute to its development and modify its associations with outcomes is crucial.

Consistent evidence indicates that renal impairment contributes to augmented long-term cardiovascular disease (CVD) risk, with cardiovascular mortality remaining the principal cause of death in this population [5,6,7,8]. Beyond its established links to coronary heart disease and heart failure, reduced kidney function may predispose individuals to atrial remodeling and AF [9,10,11]. This is plausible as kidney disease, even at early stages, can lead to activation of neurohormonal systems as Renin–Angiotensin–Aldosterone System, volume overload, oxidative stress, and systemic inflammation, which have also been implicated in atrial structural and electrical remodeling. These processes may contribute to atrial fibrosis, conduction abnormalities, and electrical instability, collectively predisposing individuals to atrial cardiopathy [12,13,14,15]. Despite this, less is known about whether reduced renal function predisposes one to atrial cardiopathy and whether their co-occurrence amplifies the risk of mortality in the general population.

We hypothesize that reduced eGFR is associated with increased risk of atrial cardiopathy, and that both conditions, whether independent or coexistent, increase the risk of mortality in the general population. Therefore, we investigated the associations of reduced eGFR with risk of atrial cardiopathy, as well as their independent and combined impact on all-cause mortality, using data from the United States Third National Health and Nutrition Examination Survey (NHANES III).

## 2. Methods and Materials

The National Health and Nutrition Examination Survey (NHANES) is an ongoing, nationally representative survey conducted by the National Center for Health Statistics (NCHS) under the Centers for Disease Control and Prevention (CDC) to assess health and disease patterns among the non-institutionalized U.S. population. NHANES-III was conducted between 1988 and 1994 with approval from the NCHS Research Ethics Review Board, and all participants provided written informed consent. Detailed descriptions of the study design and methodology have been published previously [13]. By study design, only NHANES-III participants aged ≥ 40 years underwent electrocardiogram (ECG) recording. For the present analysis, we excluded individuals with missing ECG data, non-sinus rhythm, or estimated glomerular filtration rate (eGFR) < 15 mL/min/1.73 m^2^. Additionally, we excluded those with major ECG abnormalities defined using ECG Minnesota codes (i.e., atrial fibrillation, atrial flutter, paced rhythm, complete left or right bundle branch block, or significant conduction delays) to avoid misclassification of atrial cardiopathy markers as these rhythms may alter P-wave morphology and duration.

Sociodemographic information such as age in years, sex (male, female), race/ethnicity (White, Black, Mexican American, others), smoking status (ever Smoked, never Smoked), education, family income, and medication use was obtained through structured in-home interviews. Height and weight were measured at mobile examination centers and used to calculate body mass index (BMI; kg/m^2^). Blood pressure was measured up to three times in the seated position and mean systolic and diastolic values were recorded. Diabetes mellitus was defined as fasting plasma glucose ≥ 126 mg/dL or use of glucose-lowering medication. Laboratory assays conducted by NCHS included fasting glucose, total cholesterol, and serum creatinine, among other metabolic measures.

Blood samples were collected by venipuncture during home visits or at mobile examination centers. Renal function was assessed using eGFR, calculated from serum creatinine with the Chronic Kidney Disease Epidemiology Collaboration (CKD-EPI) equation. Participants were stratified into two categories: eGFR ≥ 45 mL/min/1.73 m^2^ and eGFR < 45 mL/min/1.73 m^2^.

Atrial cardiopathy was defined by the presence of at least one of the following abnormalities on resting 12-lead ECGs: abnormal P-wave axis (outside 0–75°), deep terminal negativity in V1 (<100 µV), or prolonged P-wave duration in lead II (>120 ms). ECGs were recorded using a Marquette MAC 12 electrocardiograph (Marquette Medical Systems, Milwaukee, WI, USA) during mobile examination center assessments [14,16]. Digital ECG signals were transmitted to the Epidemiological Cardiology Research Center (EPICARE) at Wake Forest University School of Medicine (Winston-Salem, NC, USA) for central processing. All ECGs were visually inspected by trained technicians and then automatically analyzed using the GE 12-SL 2001 program (Marquette Medical Systems, Milwaukee, WI, USA).

All-cause mortality was determined through linkage with the National Death Index, with follow-up through 31 December 2015.

### Statistical Analysis

We compared participant characteristics by presence of atrial cardiopathy using Chi-square tests for categorical variables and Student’s *t*-tests for continuous variables. Categorical variables are presented as counts and percentages, and continuous variables as mean ± standard deviation (SD).

Multivariable logistic regression was used to examine the cross-sectional association between eGFR and atrial cardiopathy. eGFR was modeled both categorically (≥45 vs. <45 mL/min/1.73 m^2^) and continuously (per 1-SD decrease). Two models were constructed: Model 1 adjusted for age, sex, race/ethnicity, education, and income; Model 2 included Model 1 covariates plus prior cardiovascular disease, smoking status, diabetes, total cholesterol, lipid-lowering medication use, body mass index, systolic blood pressure, and antihypertensive medication use.

Mortality incidence rates were calculated per 1000 person-years, stratified by eGFR level and atrial cardiopathy status. Multivariable Cox proportional hazards models were used to evaluate associations of impaired renal function (eGFR < 45 vs. ≥45 mL/min/1.73 m^2^), atrial cardiopathy (present vs. absent), and their combined presence with all-cause mortality. Covariate adjustments followed the same sequence as in logistic regression (Model 1 and Model 2). The interaction between eGFR and atrial cardiopathy was tested in Model 2.

In sensitivity analysis, we ran the same adjusted models for the association between eGFR and atrial cardiopathy and their influences on CV mortality using eGFR cutoff of 60 mL/min/1.73 m^2^.

All analyses were conducted using SAS version 9.4 (SAS Institute Inc., Cary, NC, USA). Statistical significance was defined as a two-sided *p* < 0.05.

## 3. Results

This analysis included 6573 participants (mean age 57.0 ± 13.0 years; 50.5% women; 74.6% White). At baseline, 47.9% (n = 3151) had atrial cardiopathy. Compared with those without atrial cardiopathy, affected participants were older and more likely to have diabetes and a history of CVD. They also exhibited higher systolic blood pressure, greater body mass index, lower mean eGFR, and a higher proportion with eGFR < 45 mL/min/1.73 m^2^ (Table 1).

In logistic regression models adjusted for sociodemographic and cardiovascular risk factors, reduced eGFR (<45 mL/min/1.73 m^2^) was associated with significantly 44% higher odds of atrial cardiopathy compared with eGFR ≥ 45 mL/min/1.73 m^2^. When modeled as a continuous variable, each 1-SD decrease in eGFR was similarly associated with increased odds of atrial cardiopathy (OR: 1.08; 95% CI: 1.01–1.15) (Table 2). Furthermore, when looking at the association between eGFR (modeled as <60 and ≥60 mL/min/1.73 m^2^), results exhibited the same positive association but lacked statical significance (Supplement Table S1).

Over a median follow-up of 18.1 years (IQR 11.0–20.5), 3076 deaths occurred. In Cox proportional hazards models adjusted for sociodemographic and cardiovascular risk factors, both atrial cardiopathy and reduced renal function were associated with higher odds of mortality (HR: 1.12; 95% CI: 1.04–1.20) vs. (HR:1.50; 95% CI: 1.34–1.68), respectively. In a dose response fashion, each 1-SD decrease in eGFR was associated with an incremental 10% increased risk of mortality (Table 3 and Table 4). In sensitivity analysis looking at eGFR cutoff of 60 mL/min/1.73 m^2^ revealed similar results with OR of 1.30, *p* < 0.001 for the low eGFR and atrial cardiopathy group on risk of CV mortality (Supplement Table S2).

**Figure 1:**
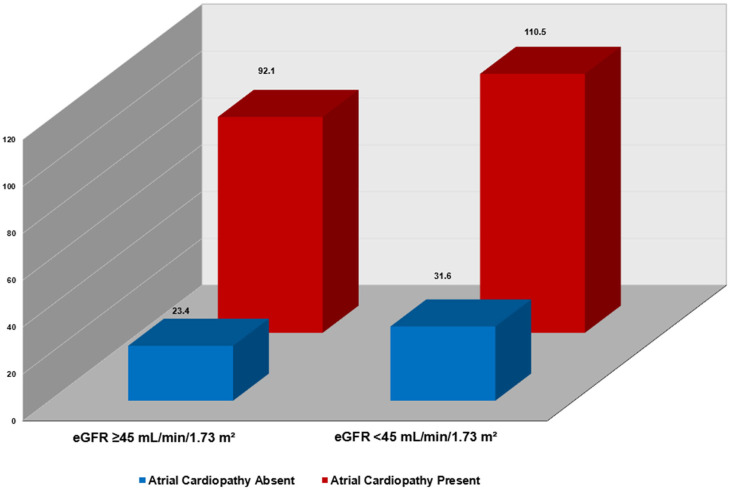
All-Cause Mortality Incidence Stratified by eGFR Levels and Atrial Cardiopathy Status. Incidence rate per 1000 person-years. eGFR: estimated glomerular filtration rate.

In joint analyses, mortality risk increased stepwise across combinations of atrial cardiopathy and eGFR categories. As illustrated in Figure 1, the highest mortality rate was observed among participants with both atrial cardiopathy and eGFR < 45 mL/min/1.73 m^2^, while the lowest rate was observed in those without atrial cardiopathy and with eGFR ≥ 45 mL/min/1.73 m^2^. In multivariable Cox models, coexistence of both conditions conferred the greatest risk (HR: 1.68; 95% CI: 1.46–1.94), exceeding the risk associated with atrial cardiopathy alone or impaired renal function defined as lower eGFR < 45 mL/min/1.73 m^2^ alone (interaction *p* = 0.011) (Table 5).

We evaluated whether the associations of atrial cardiopathy and eGFR with CV mortality varied by age, sex, or race in Table 6.

For atrial cardiopathy, there was significant effect modification by race (interaction *p* = 0.024), indicating that the strength of the association differed between Black and non-Black participants. No interaction was observed with age (*p* = 0.53) or sex (*p* = 0.77).

In contrast, for eGFR, there were significant interactions with race (*p* = 0.001) and age (*p* < 0.001). These findings indicate that the relationship between reduced kidney function and cardiovascular mortality varies across these demographic groups. No significant interaction with sex was identified (*p* = 0.18).

We further examined whether the association of the combined atrial cardiopathy–eGFR grouping with CV mortality differed by age, sex, or race. There was no evidence of interaction with age (*p* = 0.72) or sex (*p* = 0.98), indicating that the relationship between the combined atrial cardiopathy-eGFR categories and CV mortality was consistent across age groups and between men and women. However, there was a significant interaction with race (*p* = 0.004), suggesting that the CV mortality risk associated with the combined atrial cardiopathy–eGFR categories differed between Black and non-Black participants.

## 4. Discussion

In this racially and ethnically representative sample from NHANES III, our study revealed that reduced kidney function (eGFR < 45 mL/min/1.73 m^2^) is associated with increased risk of atrial cardiopathy. In longitudinal analysis, both reduced eGFR and atrial cardiopathy were independently associated with increased risk of all-cause mortality. Importantly, when both conditions coexisted, the risk of mortality was highest. This observation reflects the overlapping and synergistic mechanisms that accelerate progression toward adverse outcomes. With increasing prevalence, mortality, and morbidity of both cardiovascular and renal diseases worldwide, examining their interrelationship as well as identifying modifying factors associated with worse prognosis is crucial.

Over recent years, research has increasingly clarified the interrelationship between kidney disease and atrial remodeling, linking renal dysfunction to atrial arrhythmias, particularly AF [17,18]. In our analysis, reduced eGFR was associated with a 44% higher risk of atrial cardiopathy, underscoring the adverse impact of impaired renal function on atrial structure and electrophysiology. These findings are consistent with and extend previous reports linking renal dysfunction to atrial pathology.

For an instance, in the MESA study, participants with both electrocardiographic left atrial abnormality and albuminuria had a 2.43-fold higher risk of developing AF compared with those without either condition [17]. A large systematic review and meta-analysis by Ha et al., including over 28 million participants, further confirmed that eGFR < 60 mL/min/1.73 m^2^ and albuminuria were associated with 43% and 64% higher risks of incident AF, respectively, reinforcing the link between renal impairment and atrial electrical abnormalities [19]. Interestingly, Gong et al. also demonstrated a possible bidirectional relationship in diabetic populations, showing that left atrial volume index and strain parameters were independently associated with renal impairment, with progressive atrial dysfunction as kidney disease advanced [20]. Collectively, these findings suggest that kidney dysfunction and atrial remodeling are interrelated processes that may amplify each other’s adverse effects.

Although some of these studies evaluated AF as the primary outcome, the observed associations likely reflect a shared atrial disease substrate characterized by fibrosis, conduction delay, and mechanical dysfunction. Our findings extend this evidence by demonstrating that reduced eGFR is associated with atrial cardiopathy which is an intermediate phenotype of atrial disease representing a mechanistic pathway linking renal dysfunction to increased all-cause mortality.

The association between low eGFR and atrial cardiopathy reflects a combination of structural, electrical, and metabolic stress on the atria. Reduced kidney function promotes chronic activation of the renin–angiotensin–aldosterone and sympathetic nervous systems, leading to systemic inflammation, which in turn trigger oxidative stress, and fluid overload and impaired sodium–water excretion increase atrial stretch, promoting abnormal conduction and enlargement. Electrolyte imbalances, particularly disturbances in potassium, calcium, and phosphate may further alter atrial conduction velocity. These processes increase atrial wall tension, trigger fibroblast activation, and cause extracellular matrix deposition, resulting in atrial fibrosis and impaired conduction [21]. Alongside this, the electrolyte imbalances associated with low eGFR including elevated serum levels of potassium and phosphorus play a role as well as it disrupts ion channel function, prolong atrial depolarization, and alter P-wave morphology [22]. Over time, these changes create a vulnerable atrial substrate prone to abnormal conduction and mechanical dysfunction, even before the onset of atrial fibrillation [22,23,24].

From a prognostic standpoint, our findings demonstrated that both atrial cardiopathy and reduced kidney function are independently associated with increased mortality risk, with the greatest risk observed when both conditions coexist. This pattern supports the hypothesis that structural and electrical atrial abnormalities in atrial cardiopathy may amplify the systemic consequences of renal dysfunction, while declining kidney health may worsen atrial remodeling, together driving patients into a higher-risk trajectory.

The mortality risk associated with kidney disease is largely attributed to enhanced cardiovascular risk, even beyond progression to end-stage renal failure in many cohorts [5,6,7,8,25,26,27]. The mechanisms include a proinflammatory state, oxidative stress, endothelial dysfunction, and activation of maladaptive neurohormonal systems, which together accelerate vascular aging, atherosclerosis, vascular calcification, and myocardial fibrosis [21,22,23,24,28,29]. Impaired excretory function leads to accumulation of uremic toxins, volume overload, disturbances in mineral metabolism, and electrolyte derangements, all of which further stress the cardiovascular system [22]. These pathologies can impair myocardial structure, promote left ventricular hypertrophy, and predispose to arrhythmias and sudden cardiac death [25,26]. In addition, microvascular ischemia and endothelial injury inherent to kidney disease may likewise contribute to atrial ischemia and fibrosis, setting the stage for atrial cardiopathy [27].

Meanwhile, atrial cardiopathy confers mortality risk through mechanisms linked to thrombosis, arrhythmia, and heart failure [28,29]. Recent work further highlights that atrial cardiomyopathy represents a progressive substrate that predisposes patients to AF [30]. Structural and electrical remodeling of the atria including fibrosis and conduction heterogeneity promotes blood stasis, which increases the likelihood of clot formation and systemic embolism [31]. In addition, the effect of atrial cardiopathy on atrial structural and functional status can lead to atrial contractile dysfunction, thereby reducing cardiac output and promoting heart failure decompensation [28]. Several cohort studies have shown that markers of atrial dysfunction as evident by abnormal P-wave indices or reduced atrial strain predict all-cause mortality and ischemic events independent of overt AF [31].

The observed synergy between reduced eGFR and atrial cardiopathy may reflect the intersection of these shared and distinct pathways. Systemic inflammation, oxidative stress, neurohormonal activation, endothelial injury, and fibrotic remodeling act as common mediators [21,22,23,24,28,29]. Kidney disease may potentiate atrial injury via volume overload, hypertension, metabolic derangements, and microvascular ischemia, while atrial pathology may worsen renal hemodynamics, elevate upstream pressures, compromise cardiac–renal coupling, and amplify neurohormonal stress [21,24]. The cycle may intensify over time, accelerating progression toward decompensated heart failure, arrhythmic events, and multiorgan dysfunction, thereby elevating mortality beyond what either condition would produce in isolation.

When looking at subgroups analysis, we observed deferential effect modification by age, sex and race in the association between atrial cardiopathy, eGFR levels and CV mortality. While effect modification for the CV mortality risk associated with atrial cardiopathy effect was influenced by race, associations with eGFR were mainly influenced by race and age. These results highlight the need for further studies exploring the specific interactions of atrial cardiopathy and kidney disease to provide more precise risk stratification strategies. 

Our results highlight the need for closer clinical attention to atrial cardiopathy markers in patients with reduced kidney function, as their coexistence may identify a subgroup at particularly high risk. The ECG markers may help identify CKD patients who are at higher risk of atrial fibrillation, stroke, or heart failure, even before clinical arrhythmia becomes apparent. Incorporating these markers into routine evaluation could allow earlier risk stratification, closer monitoring, and possibly earlier preventive interventions. Thus, ECG-based detection of atrial cardiopathy may offer a simple, low-cost tool to guide clinical decision-making in CKD. Understanding this overlap may help guide risk categorization and early intervention strategies, particularly in community-based settings where comorbidities may otherwise remain undetected. Clinically, our findings emphasize the importance of incorporating simple ECG markers into risk stratification for patients with impaired renal function. Identification of atrial cardiopathy in this population could inform targeted early interventions, closer cardiovascular monitoring, and refinement of existing prognostic tools to improve outcome prediction and patient management.

Furthermore, while ECG markers remain widely available and reproducible, their diagnostic performance may be enhanced by integrating additional imaging modalities and circulating biomarkers, such as NT-proBNP, high-sensitivity troponins, or Galectin-3, which can better reflect underlying structural remodeling. Recent systematic reviews confirm that NT-proBNP and Galectin-3 are independently associated with atrial fibrosis and adverse cardiovascular outcomes, reinforcing their role as complementary biomarkers for atrial cardiopathy detection [32,33]. Combining these biomarkers with ECG parameters may enable earlier detection of atrial cardiomyopathy and improve prediction of downstream complications such as atrial fibrillation and stroke. In addition, emerging evidence demonstrates that deep learning models applied to standard 12-lead ECGs can accurately predict left atrial volumes and ejection fraction, outperforming conventional ECG markers and improving risk prediction for AF, cardioembolic stroke, and heart failure [34]. This AI-based approach offers a scalable, low-cost tool for identifying atrial cardiopathy in high-risk populations, including those with CKD, where advanced imaging is often impractical. Future studies should explore the additive value of such biomarker- and AI-based frameworks in patients with CKD to optimize screening and risk stratification.

Given the synergistic risk associated with reduced eGFR and atrial cardiopathy, there is a potential clinical opportunity to intervene early in this high-risk population. Strict blood pressure and glucose control, intensive dyslipidemia therapy [35,36], and customized anticoagulant choices for specific patients are examples of therapeutic approaches [37]. Furthermore, early detection of atrial remodeling may assist direct preventative measures, such as targeted medication or lifestyle changes. These interventions may ultimately reduce the complications such as AF and ischemic stroke, though prospective studies are needed to confirm the effectiveness of such approaches in this specific subgroup..

Beyond risk stratification, pharmacological interventions represent a critical opportunity to modify the synergistic risk posed by impaired renal function and atrial cardiopathy. In patients with CKD, especially those on dialysis, the choice and dosing of medications can significantly influence atrial remodeling and cardiovascular outcomes. Agents targeting the renin–angiotensin–aldosterone system (RAAS), such as ACE inhibitors and ARBs, may attenuate atrial fibrosis and neurohormonal activation, thereby reducing the substrate for atrial cardiopathy [25,35]. Beta-blockers and certain calcium channel blockers can modulate sympathetic overactivity and improve atrial conduction stability, while statins may exert pleiotropic anti-inflammatory effects that indirectly benefit atrial health [36]. In CKD patients, the pharmacokinetics of anticoagulants are profoundly altered; for instance, warfarin or direct oral anticoagulants require individualized strategies to balance thromboembolic and bleeding risks [37]. Despite that, the ARCADIA trial found no significant reduction in recurrent stroke with apixaban compared to aspirin among patients with biomarker-defined atrial cardiopathy, indicating that atrial cardiopathy alone may not justify anticoagulation in the absence of atrial fibrillation [38]. Notably, individuals with advanced chronic kidney disease were excluded, highlighting the need for future studies to determine whether anticoagulation strategies in patients with impaired renal function or on dialysis could yield different outcomes.

Moreover, emerging therapies such as sodium-glucose cotransporter-2 (SGLT2) inhibitors and non-steroidal mineralocorticoid antagonists may offer dual renal and cardiac protection, though evidence in advanced CKD remains limited [25]. Optimizing these pharmacological approaches, alongside dialysis-related adjustments in electrolyte management, has the potential to mitigate atrial instability and improve long-term survival in this high-risk population. Future studies should explore whether integrated pharmacotherapy tailored to CKD stage and atrial cardiopathy markers can reduce mortality and cardiovascular complications.

Recent evidence highlights left atrial (LA) strain and derived indices like the LA volumetric–mechanical coupling index (LACI) as advanced biomarkers by incorporating mechanical and volumetric dysfunction that strongly predict cardiovascular events and mortality in CKD patients on dialysis [39,40,41]. These measures provide refined assessment of atrial cardiopathy beyond traditional imaging, aligning with our findings. Meta-analyses have further demonstrated that LA strain (indicative of atrial disfunction) predicts adverse outcomes independent of atrial fibrillation and LV function [42].

## 5. Limitations

Several limitations should be acknowledged. First, we adjusted many sociodemographic and cardiovascular risk factors, but some residual confounding from unmeasured variables such as inflammatory markers, medication adherence, or other socioeconomic determinants. Second, we used ECG markers for detecting the atrial cardiopathy, which, despite being reproducible and widely available, may not fully detect the structural and functional atrial remodeling that could be assessed with imaging or biomarker data. Third, excluding individuals with non-sinus rhythm or incomplete ECG data may have introduced selection bias by limiting the sample to healthier individuals, potentially underestimating the true burden of atrial cardiopathy in chronic kidney disease populations. Fourth, the association for eGFR and risk of atrial cardiopathy was done through cross-sectional analysis which limit establishment of the temporal sequence and causal inferences cannot be made. Finally, as our analysis is based on U.S. adults, generalizability to other international populations may be limited.

## 6. Conclusions

In this nationally representative cohort of middle-aged and older U.S. adults, impaired renal function and electrocardiographic atrial cardiopathy were each independently associated with increased all-cause mortality, and their coexistence conferred a synergistically elevated risk. These findings underscore the value of simple ECG markers for identifying high-risk individuals with chronic kidney disease in community settings. Incorporating atrial cardiopathy assessment into routine care for patients with reduced eGFR may enhance risk stratification and guide early preventive interventions. However, the observational nature of the study limits its interpretation. Future prospective and interventional studies are required to validate this synergistic association and to explore whether early identification and targeted intervention can improve outcomes. Such evidence would be critical to inform the development of clinical guidelines for risk stratification and management in this high-risk population.

## Figures and Tables

**Table 1:** Baseline Characteristics of the Study Participants.

Characteristics [Mean ± Standard deviation or N (%)]	Atrial Cardiopathy—Absent (n = 3422)	Atrial Cardiopathy—Present (n = 3151)	*p*-Value
Age (years)	58.2 ± 13.1	61.7 ± 13.5	<0.001
Women	1867 (54.6%)	1451 (46.1%)	<0.001
Race/Ethnicity			
White	1782 (52.1%)	1632 (51.8%)	<0.001
Black	661 (19.3%)	785 (24.9%)	
Mexican American	822 (24.0%)	643 (20.4%)	
Others	157 (4.6%)	91 (2.9%)	
Smoking Status			
Ever Smoked	1894 (55.4%)	1806 (57.3%)	0.108
Never Smoked	1528 (44.7%)	1345 (42.7%)	
Body Mass Index (kg/m^2^)	27.3 ± 5.3	28.1 ± 5.6	<0.001
Systolic Blood Pressure (mm Hg)	131.4 ± 26.4	135.9 ± 30.8	<0.001
Diastolic Blood Pressure (mm Hg)	76.7 ± 26.0	77.5 ± 27.2	0.241
Prior CVD *	258 (7.5%)	392 (12.4%)	<0.001
Use of BP Medications	674 (19.7%)	902 (28.6%)	<0.001
Diabetes Mellitus	488 (14.3%)	539 (17.1%)	0.002
Use of lipid lowering	160 (4.7%)	160 (5.1%)	0.449
Total Cholesterol (mg/dL)	225.5 ± 154.9	223.1 ± 45.3	0.382
eGFR (mean)	71.0 ± 15.6	67.4 ± 66.8	<0.001
eGFR < 45 mL/min/1.73 m^2^ (%)	161 (4.7%)	294 (9.3%)	<0.001

eGFR: estimated glomerular filtration rate. CVD: cardiovascular disease. BP: Blood pressure. * A composite of history of prior stroke, HF, and CHD.

**Table 2:** Association of eGFR levels with Atrial Cardiopathy.

eGFR Level	Atrial Cardiopathy N (%)		Model 1		Model 2	
	Present	Absent	OR (95% CI)	*p*-Value	OR (95% CI)	*p*-Value
eGFR ≥ 45 mL/min/1.73 m^2^	2857 (90.7%)	3261 (95.3%)	Reference	--	Reference	--
eGFR < 45 mL/min/1.73 m^2^	294 (9.3%)	161 (4.7%)	1.531 (1.242–1.890)	<0.001	1.442 (1.16–1.78)	<0.001
eGFR per 1-SD decrease *	--		1.112 (1.043–1.191)	0.004	1.087 (1.01–1.15)	0.0191

eGFR: estimated glomerular filtration rate. OR: odds ratio. CI: confidence interval. Model 1 adjusted for age, sex, race (Whites vs. non-whites), education (less than high school vs. other) and income ($20 k per year). Model 2 adjusted for model 1 plus smoking status (ever smoked vs. never), history of diabetes, total cholesterol, use of lipid lowering, smoking, prior CVD, body mass index, systolic blood pressure, and use of BP medication. * eGFR SD = 16.2 mL/min/1.73 m^2^.

**Table 3:** Association Between Atrial Cardiopathy and All-Cause Mortality.

Outcome	Participants N (%)	Model 1		Model 2	
		HR (95% CI)	*p*-Value	HR (95% CI)	*p*-Value
Atrial Cardiopathy-Absent	3422 (52.1)	Reference	--	Reference	--
Atrial Cardiopathy-Present	1669 (47.9)	1.140 (1.061–1.221)	<0.001	1.121 (1.043–1.202)	0.003

eGFR: estimated glomerular filtration rate. HR: hazard ratio. CI: confidence interval. Model 1: Age, sex, race, education and income. Model 2: Model 1 plus prior cardiovascular disease, smoking status, history of diabetes, total cholesterol, use of lipid lowering, smoking, body mass index, systolic blood pressure, and use of blood lowering medication.

**Table 4:** Association Between eGFR and All-Cause Mortality.

eGFR Level	All-Cause Mortality n (%)		Model 1		Model 2	
	Present	Absent	OR (95% CI)	*p*-Value	OR (95% CI)	*p*-Value
eGFR ≥ 45 mL/min/1.73 m^2^	2668 (43.6%)	N (%)	Reference	--	Reference	--
eGFR < 45 mL/min/1.73 m^2^	408 (89.7%)	N (%)	1.571 (1.410–1.766)	<0.001	1.501 (1.339–1.685)	<0.001
eGFR per 1-SD decrease *	--		1.103 (1.054–1.155)	<0.001	1.104 (1.051–1.153)	<0.001

eGFR: estimated glomerular filtration rate. OR: odds ratio. CI: confidence interval. Model 1 adjusted for age, sex, race (Whites vs. non-whites), education (less than high school vs. other) and income (< vs. >$20 k per year). Model 2 adjusted for model 1 plus CVD, smoking status (ever smoked vs. never), diagnosis of diabetes, total cholesterol, use of lipid lowering, smoking, prior CVD, body mass index, systolic blood pressure, and use of BP medication. * eGFR SD = 16.2 mL/min/1.73 m^2^.

**Table 5:** Association of Combinations of eGFR Levels and Atrial Cardiopathy Status with Mortality.

Atrial Cardiopathy and eGFR Levels	Participants (n)/Events (%)	Model 1		Model 2	
		HR (95% CI)	*p*-Value	HR (95% CI)	*p*-Value
Atrial cardiopathy Absent + normal eGFR	3261/1267 (38.9%)	Reference	--	Reference	--
Atrial cardiopathy Absent + reduced eGFR	2857/1401 (49.0%)	1.121 (1.044–1.212)	0.003	1.101 (1.022–1.183)	0.0190
Atrial cardiopathy present + normal eGFR	161/140 (87.0%)	1.550 (1.302–1.863)	<0.001	1.421 (1.18–1.70)	<0.001
Atrial cardiopathy present + reduced eGFR	294/268 (91.2%)	1.752 (1.521–2.011)	<0.001	1.681 (1.460–1.940)	<0.001

eGFR = estimated glomerular filtration rate. HR: hazard ratio. CI: confidence interval. Model 1 adjusted for age, sex, race/ethnicity, education and income. Model 2 adjusted for model 1 plus diabetes, total cholesterol, use of lipid lowering medications, smoking status, history of prior cardiovascular disease, body mass index, systolic blood pressure, and use of blood pressure medications. Interaction *p*-value for eGFR x atrial cardiopathy calculated using model 2 covariates = 0.011.

**Table 6:** Association of Combinations of eGFR Levels and Atrial Cardiopathy Status with Mortality in Subgroups.

Subgroup	Atrial Cardiopathy/eGFR Group	Model 1 HR (95% CI)	Model 2 HR (95% CI)
Women	Both absent (reference)	Ref.	Ref.
	Only atrial cardiopathy	1.102 (0.952–1.282)	1.073 (0.925–1.255)
	Only eGFR < 60	1.101 (0.932–1.293)	1.08 (0.925–1.277)
	Both Present	1.371 (1.172–1.611)	1.302 (1.104–1.520)
Men	Both absent (reference)	Ref.	Ref.
	Only atrial cardiopathy	1.051 (0.932–1.195)	1.04 (0.922–1.177)
	Only eGFR < 60	1.074 (0.912–1.274)	1.05 (0.894–1.247)
	Both Present	1.313 (1.132–1.513)	1.31 (1.134–1.514)
Non-Black	Both absent (reference)	Ref.	Ref.
	Only atrial cardiopathy	0.971 (0.791–1.183)	1.000 (0.811–1.224)
	Only eGFR < 60	0.920 (0.730–1.183)	0.940 (0.742–1.203)
	Both Present	1.302 (1.057–1.629)	1.321 (1.062–1.652)
Blacks	Both absent (reference)	Ref.	Ref.
	Only atrial cardiopathy	1.093 (0.981–1.210)	1.053 (0.943–1.172)
	Only eGFR < 60	1.123 (0.981–1.272)	1.091 (0.966–1.247)
	Both Present	1.335 (1.188–1.503)	1.284 (1.144–1.456)
<65 years	Both absent (reference)	Ref.	Ref.
	Only atrial cardiopathy	1.022 (0.891–1.162)	0.986 (0.856–1.122)
	Only eGFR < 60	1.132 (0.891–1.439)	1.123 (0.891–1.431)
	Both Present	1.571 (1.272–1.953)	1.377 (1.100–1.712)
≥65 years	Both absent (reference)	Ref.	Ref.
	Only atrial cardiopathy	1.102 (0.97–1.26)	1.091 (0.953–1.240)
	Only eGFR < 60	1.062 (0.923–1.211)	1.050 (0.920–1.211)
	Both Present	1.289 (1.122–1.454)	1.284 (1.134–1.464)

eGFR: estimated glomerular filtration rate. For atrial cardiopathy group, interaction *p* for age, sex and race (0.53, 0.77, 0.024), respectively. For eGFR <60 group, interaction *p* for age, sex and race (<0.001, 0.18, 0.001), respectively. For the combined atrial cardiopathy–eGFR group “Both Present” interaction *p* for age, sex and race (0.72, 0.98, 0.004), respectively. Analysis conducted for the association between each group and risk for CV mortality.

## Data Availability

Data used in this study are publicly available at https://wwwn.cdc.gov/nchs/nhanes/nhanes3/datafiles.aspx, accessed on 1 March 2023.

## Supplementary Materials

The following supporting information can be downloaded at https://www.mdpi.com/article/10.3390/jcm15010122/s1, Table S1: Association of eGFR levels (<60 and ≥60 mL/min/1.73 m^2^)with Atrial Cardiopathy; Table S2: Atrial Cardiopathy/eGFR levels (levels (<60 and ≥60 mL/min/1.73 m^2^) Combinations and CV Mortality.
