# Supplementary material for: Renal Function, Atrial Cardiopathy, and Their Joint Association with Mortality in the General Population
Source: J Clin Med. 2025 Dec 24;15(1):122. doi: 10.3390/jcm15010122 (PMC12786734; doi:10.3390/jcm15010122)
Supplement: Supplementary file 1 [file jcm-15-00122-s001.zip › jcm-3995712-supplementary.pdf]

Manuscript: **jcm-3995712**: Renal Function, Atrial Cardiopathy, and Their Joint Association with Mortality in the General Population

Supplementary materials

Supp. Table S1. Association of eGFR levels (<60 and ≥60 mL/min/1.73 m²)with Atrial Cardiopathy .

Supp. Table S2: Atrial Cardiopathy/eGFR Combinations and CV Mortality

Supp. Table S1. Association of eGFR levels (<60 and ≥60 mL/min/1.73 m^2)with Atrial Cardiopathy .

eGFR Level	Model 1		Model 2	
	OR (95% CI)	p-value	OR (95% CI)	p-value
eGFR ≥60 mL/min/1.73 m^2	Reference	--	Reference	--
eGFR <60 mL/min/1.73 m^2	1.14(1.00 – 1.29)	0.0475	1.07(0.94-1.22;)	0.300

eGFR: estimated glomerular filtration rate. OR: odds ratio. CI: confidence interval.

Model 1 adjusted for age, sex, race (Whites vs. non-whites), education (less than high school vs. other) and income ($20k per year).

Model 2 adjusted for model 1 plus smoking status (ever smoked vs never), history of diabetes, total cholesterol, use of lipid lowering, smoking, prior CVD, body mass index, systolic blood pressure, and use of BP medication.

Supp. Table S2: Atrial Cardiopathy/eGFR Combinations and CV Mortality

**Atrial Cardiopathy/eGFR**	**Model 1**<br>**HR (95% CI)**	**p-value**	**Model 2**<br>**HR (95% CI)**	**p-value**
Both absent	Ref	--	--	--
Only Atrial Cardiopathy	1.07 (0.98–1.18)	0.15	1.05 (0.95–1.15)	0.35
Only eGFR <60	1.08 (0.96–1.21)	0.18	1.07 (0.95–1.20)	0.28
Both present	1.33 (1.19–1.48)	<0.001	1.30 (1.16–1.45)	<0.001

eGFR= estimated glomerular filtration rate. HR: hazard ratio. CI: confidence interval.

Model 1 adjusted for age, sex, race/ethnicity, education and income.

Model 2 adjusted for model 1 plus diabetes, total cholesterol, use of lipid lowering medications, smoking status, history of prior cardiovascular disease, body mass index, systolic blood pressure, and use of blood pressure medications.
